# Exploratory Metabolomic Analysis Based on Reversed-Phase Liquid Chromatography–Mass Spectrometry to Study an In Vitro Model of Hypoxia-Induced Metabolic Alterations in HK-2 Cells

**DOI:** 10.3390/ijms22147399

**Published:** 2021-07-09

**Authors:** Samuel Bernardo-Bermejo, Elena Sánchez-López, Lei Tan, Selma Benito-Martínez, Zhengjin Jiang, María Castro-Puyana, Francisco Javier Lucio-Cazaña, María Luisa Marina

**Affiliations:** 1 Departamento de Química Analítica, Química Física e Ingeniería Química, Universidad de Alcalá, Ctra. Madrid-Barcelona Km. 33.600, 28871 Madrid, Spain; samuel.bernardo@edu.uah.es (S.B.-B.); lei.tan@edu.uah.es (L.T.); maria.castrop@uah.es (M.C.-P.); 2 Department of Human Genetics, Leiden University Medical Center, 2333 ZA Leiden, The Netherlands; e.sanchez_lopez@lumc.nl; 3 Institute of Pharmaceutical Analysis, College of Pharmacy, Jinan University, Guangzhou 510632, China; jzjjackson@hotmail.com; 4 Departamento de Biología de Sistemas, Universidad de Alcalá, Ctra. Madrid-Barcelona Km. 33.600, 28871 Madrid, Spain; selma.benito@uah.es (S.B.-M.); javier.lucio@uah.es (F.J.L.-C.); 5 “Ramón y Cajal” Health Research Institute (IRYCIS), Universidad de Alcalá, 28871 Madrid, Spain; 6 Instituto de Investigación Química Andrés M. del Río, Universidad de Alcalá, Ctra. Madrid-Barcelona Km. 33.600, 28871 Madrid, Spain

**Keywords:** HK-2 cells, hypoxia, liquid chromatography–mass spectrometry, untargeted metabolomics, multivariate analysis

## Abstract

Oxygen deficiency in cells, tissues, and organs can not only prevent the proper development of biological functions but it can also lead to several diseases and disorders. In this sense, the kidney deserves special attention since hypoxia can be considered an important factor in the pathophysiology of both acute kidney injury and chronic kidney disease. To provide better knowledge to unveil the molecular mechanisms involved, new studies are necessary. In this sense, this work aims to study, for the first time, an in vitro model of hypoxia-induced metabolic alterations in human proximal tubular HK-2 cells because renal proximal tubules are particularly susceptible to hypoxia. Different groups of cells, cultivated under control and hypoxia conditions at 0.5, 5, 24, and 48 h, were investigated using untargeted metabolomic approaches based on reversed-phase liquid chromatography–mass spectrometry. Both intracellular and extracellular fluids were studied to obtain a large metabolite coverage. On the other hand, multivariate and univariate analyses were carried out to find the differences among the cell groups and to select the most relevant variables. The molecular features identified as affected metabolites were mainly amino acids and Amadori compounds. Insights about their biological relevance are also provided.

## 1. Introduction

Oxygen, one of the most abundant elements in the atmosphere, is essential for carrying out the cellular function successfully. While an excessive supply of oxygen (hyperoxia) makes it a highly toxic molecule [1], insufficient oxygen availability (hypoxia) can lead to serious consequences for all living beings [2]. Thus, maintaining an adequate oxygen level is a key factor. Hypoxia results in hyperventilation and sympathetic activation in mammals, increasing the uptake and distribution of oxygen to cells and tissues within a few seconds. On the other hand, the generation of new blood vessels and the synthesis of erythrocytes and nonaerobic ATP increase if the hypoxia occurs for hours or days [1]. To carry out these adaptations, an extensive genetic program is necessary, which is possible thanks to the hypoxia-inducible factors (HIFs) [3]. HIFs are transcription factors composed of an oxygen-sensitive subunit (HIF-1α or HIF-2α) and an oxygen-insensitive subunit (HIF-1β), the former being strictly tuned by the oxygen tension [4]. HIF-1α is ubiquitously expressed and is found in most renal epithelial cells, including proximal tubular cells [3].

In recent years, several studies based on hypoxia have demonstrated its influence on the development and progression of different diseases and disorders related to the kidney. In this sense, researchers have tried to dissect the possible molecular mechanisms involved in these conditions [5]. However, the hypoxia function in the origin and development of these diseases is not completely understood and it is important to develop new studies that allow a better understanding. For instance, although the initiators of diabetic kidney disease are not well established, Vinokvskis et al. highlighted the importance of renal hypoxia as an early phenomenon in diabetes that leads to this disorder [6]. In addition, different researchers support the idea of hypoxia as a marker of poor renal function and, as a consequence of insufficient oxygen supply, a cellular response takes place [4].

Metabolomics is the omic science closest to phenotype, which provides relevant information about the metabolome derived from tissues, cells, biofluids, or a specific organism, especially the untargeted approach. Thus, this science helps us to dissect the molecular mechanisms that cause different disorders and to understand their pathological process. Metabolomics employs different advanced analytical techniques such as liquid chromatography–mass spectrometry (LC–MS), gas chromatography–mass spectrometry (GC–MS), capillary electrophoresis–mass spectrometry (CE–MS), and nuclear magnetic resonance (NRM) to analyze as many metabolites as possible (polar and non-polar compounds) to cover the whole metabolome. Among these techniques, LC–MS is the most used due to its advantages such as its ability to analyze underivatized samples, and its great robustness and elevated sensitivity [7].

Cell metabolomics studies have increased over time since they can shed light on a myriad of different biological questions. Cells have important advantages when compared to other samples. They show low biological variability because they can be cultured in more controlled experimental conditions and they are also normally less expensive, raise no/low ethical concerns, and provide valuable information which is usually easier to interpret [8,9].

Cell metabolomics studies based on hypoxia have been mainly carried out in cancer cell lines [10,11,12,13,14] but, as far as we know, no report exits employing metabolomics analysis in an in vitro study of hypoxia at normal glucose levels using HK-2 cells. In previous works carried out by our group, the metabolites affected by high glucose in an in vitro model of HK-2 cells, which contributed to unveil the molecular mechanisms of diabetic nephropathy, were investigated [15,16]. The results obtained in those works demonstrated that this human proximal tubular cell line can be considered a good candidate for studying the high glucose effect as well as the genesis and development of diabetic nephropathy. Recently, Valdés et al. investigated the metabolic changes in HK-2 cells exposed simultaneously to high glucose (25 mM) and hypoxia (1% O_2_) [17]. However, the choice of evaluating combined hyperglycemia with hypoxia hampered the effects caused only by hypoxia.

Thus, the aim of this work was to carry out the first metabolomic study on HK-2 cells, focusing solely on hypoxia effects on the metabolism by culturing these cells at different hypoxic times. To obtain a large metabolite coverage, both intracellular and extracellular fluids were analyzed. The present work provides insights about metabolic changes associated with hypoxia in a renal context.

## 2. Results and Discussion

### 2.1. Untargeted Metabolomics Analysis of an In Vitro Model of Hypoxia in HK-2 Cells

#### 2.1.1. Metabolomic Analysis at Hypoxia Times of 0.5 and 5 h (Short-Term Hypoxia)

**Figure 1:**
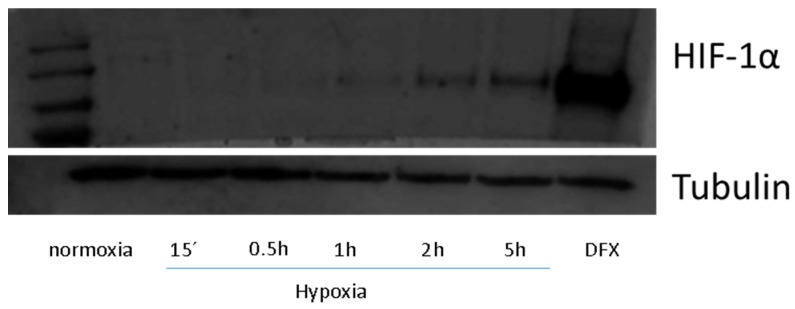
Effect of short-term hypoxia (Western Blot analysis) on the expression of HIF-1α in HK-2 cells. DFX, desferrioxamine.

Hypoxia can take place both under physiological and pathological conditions. Herein, we wanted to study the changes occurring at the metabolome level in vitro. To select the hypoxia time that is more suitable for inducing sufficient metabolic changes in HK-2 cells, the expression of HIF-1α was assayed over different time points. As can be seen in Figure 1, HIF-1α starts to show increased expression at times longer than 1 h, which suggests that hypoxia-like conditions might have been triggered. However, the accumulation of HIF-1α is far from reaching its maximal value even after 5 h under hypoxia (compared with the expression of HIF-1α found in cells treated with desferrioxamine (DFX), which fully prevents proteasome-dependent degradation of HIF-1α). To evaluate whether metabolic changes were already produced at this early stage, metabolic analyses of cells cultured under hypoxia for 0.5 h (HIF-1α not considerably expressed) and 5 h (HIF-1α more expressed) were performed. To do that, five groups of HK-2 cells were established: cells under control conditions for 0 h (Ct0), 0.5 h (Ct0.5h) and 5 h (Ct5h), and cells under hypoxia conditions during 0.5 h (Ht0.5h) and 5 h (Ht5h).

To obtain a more comprehensive view, both endometabolome and exometabolome were analyzed using LC–MS in the reversed phase (RPLC) mode (ESI+ mode), as previously developed [15]. Each metabolomic sequence consisted of five sample groups, which were formed by five biological replicates and each of them was analyzed three times (a total of 75 sample injections). The quality control (QC) was also analyzed throughout the sequence (see Section 3.6) a total of 21 times. Data obtained in each of the two sequences were processed according to Section 3.7. Once the feature alignment and filtering were carried out, a total of 55 and 175 metabolic features were obtained from intracellular fluid and extracellular fluid, respectively.

**Figure 2:**
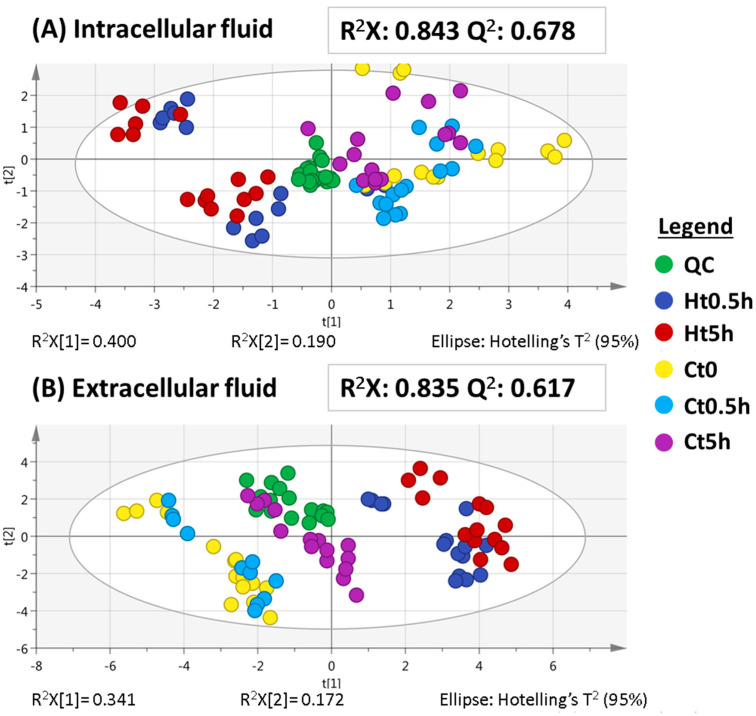
PCA for both intracellular fluid (**A**) and extracellular fluid (**B**) sequences at short times. Hotelling’s ellipses represent the 95% confidence limit. QC, quality control. Ht0.5h, hypoxia at 0.5 h. Ht5h, hypoxia at 5 h. Ct, control at 0 h. Ct0.5h, control at 0.5 h. Ct5h, control at 5 h.

As a consequence of the complexity of the generated datasets, multivariate statistical analysis was chosen since it is a very resourceful option for these types of exploratory studies. In this sense, to study the important metabolic changes among the five groups, unsupervised principal components analysis (PCA) was performed (Figure 2). Data normalization is a critical step in every metabolomic study to reduce possible systematic errors and to be able to properly compare the data. Although normalization using protein content is one of the most common approaches, it might not be the best approach in this case since hypoxia might alter protein production and thus bias the results [18]. Thus, herein we normalized the data using the number of cells per group sample, as detailed in the experimental part.

Figure 2 shows the PCA score plots of the intracellular and extracellular analyses. In both cases, QC samples appeared clustered, especially in the case of the intracellular sequence (Figure 2A), which highlights the success of the performance of both runs. The relatively worse cluster in the QC, seen in the extracellular fluid (Figure 2B), might be due to a minor analytical drift but it is acceptable since the variability is significantly lower than that of the experimental groups. The consistency and robustness of both sequences were demonstrated when the PCA, including the QC samples, was compared to the PCA models excluding QCs from the PCA models and significant changes were not observed (data not shown).

Taking a closer look at this unbiased analysis, in both fluids there was a clear difference between the hypoxic and control groups, and both showed high R^2^X and Q^2^ values. The clusters observed per experimental group, according to whether the cells were at hypoxic or control conditions, reveal that there are noticeable changes in their respective metabolomes.

To discover the most significant alterations among the experimental groups in the two fluids, supervised analysis was then carried out comparing the hypoxia vs. the control at the same time, in order to eliminate time-dependent bias. Two partial least squares-discriminant analyses (PLS–DA) were carried out for Ht0.5h vs. Ct0.5h and Ht5h vs. Ct5h groups. PLS–DA score plots showed acceptable R^2^X, R^2^Y, and Q^2^ parameters for both fluids, and whereas R^2^X values are slightly higher in the intracellular fluid, R^2^Y and Q^2^ are slightly higher in the extracellular fluid, as can be seen in Table 1. In this table, the F- and *p*-values obtained from the cross-validated ANOVA (CV-ANOVA) are included and it high F- and low *p*-values can be observed in all cases generally but the extracellular fluid shows even better results. For validating the PLS–DA models, a permutation test using a total of 200 permutations was conducted (Figure S1). This test allowed us to confirm that there are statistically significant differences between the studied pairs, that is, Ht0.5h vs. Ct0.5h and Ht5h vs. Ct5h groups. In general, the extracellular fluid showed more important differences than intracellular fluid, which strengthens the relevance of studying the exometabolome, although most of the time this fluid is often discarded in metabolomics studies [8].

Once the differences between the groups were established, all molecular features with a variable importance in the projection (VIP) value higher than 1.00 in Ht0.5h vs. Ct0.5h and Ht5h vs. Ct5h were selected in both fluids to find those variables affected by hypoxia at these times. In this sense, a total of 20 and 47 statistically significant features were obtained from intracellular and extracellular fluid of HK-2 cells, respectively. Table 2 and Table S1 show the values of retention time, molecular formula, metabolite, identification level, monoisotopic mass, the error accuracy compared to the databases, the main fragments retrieved from the MS/MS spectra, the VIP values for the PLS–DA models, and the trend for all the significant variables.

In the intracellular fluid, there were three significant variables at 0.5 h, six significant variables at 5 h and 11 eleven significant variables were common at 0.5 and 5 h. According to the Metabolomics Standard Initiative (MSI) guidelines [19], the metabolites butyrylcarnitine and pyroglutamic acid at 5 h, and acetylcarnitine and pyridoxine, which were common at 0.5 h and 5 h, were identified as level 1.

In the extracellular fluid, there were five significant variables at 0.5 h, 17 significant variables at 5 h, and 25 significant variables were common at 0.5 and 5 h. Phenylacetylglycine was identified as level 1 at 0.5 h whereas the Amadori compounds N-(1-deoxy-1-fructosyl)leucine or N-(1-deoxy-1-fructosyl)isoleucine at 5 h and 4-Hydroxy-3-methoxy-a-methylphenylalanine and thyronine, which was common at 0.5 and 5 h, were identified as level 2. The Amadori compounds were identified by corresponding their MS/MS spectra to those reported in the literature [20], whereas L-4-hydroxy-3-methoxy-a-methylphenylalanine and thyronine could be identified due to their main fragmentations corresponding to those that were found in the predicted MS/MS spectra from the METLIN database (https://metlin.scripps.edu) and CFM-ID (cfmid.wishartlab.com), respectively (both accessed on 1 April 2021).

In both cases, it can be observed that the number of affected metabolites is higher at 5 h and also that in the extracellular fluid the number was higher than in the intracellular fluid. Although at these times the hypoxia could start to induce changes at the molecular level, it seems that the metabolome of HK-2 cells is not affected by great changes. In this way, and as a consequence of the obtained results, an additional metabolomic analysis was achieved using longer hypoxia times.

#### 2.1.2. Metabolomic Analysis at Hypoxia Times of 24 and 48 h (Long-Term Hypoxia)

**Figure 3:**
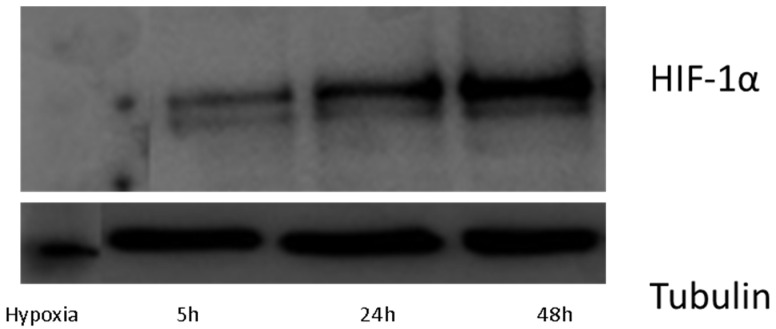
Time-course of the expression of HIF-1α (Western Blot analysis) of HK-2 cells: Comparison between the effect of hypoxia at 5 h and longer times.

To study the hypoxia effect at longer times, a second metabolomic sequence was carried out in a similar way to the previous one. This time, HK-2 cells were subjected to 24 and 48 h. Figure 3 shows the expression of HIF-1α at longer times where they are higher compared to the short times. Thus, the new experimental design included HK-2 cells under control conditions during 0 h (Ct0), 24 h (Ct24h) and 48 h (Ct48h), and cells under hypoxia conditions during 24 h (Ht24h) and 48 h (Ht48h). To illustrate what the LC analyses looked like, Figure S2 shows the total ion chromatogram of control and hypoxia samples at 48 h.

**Figure 4:**
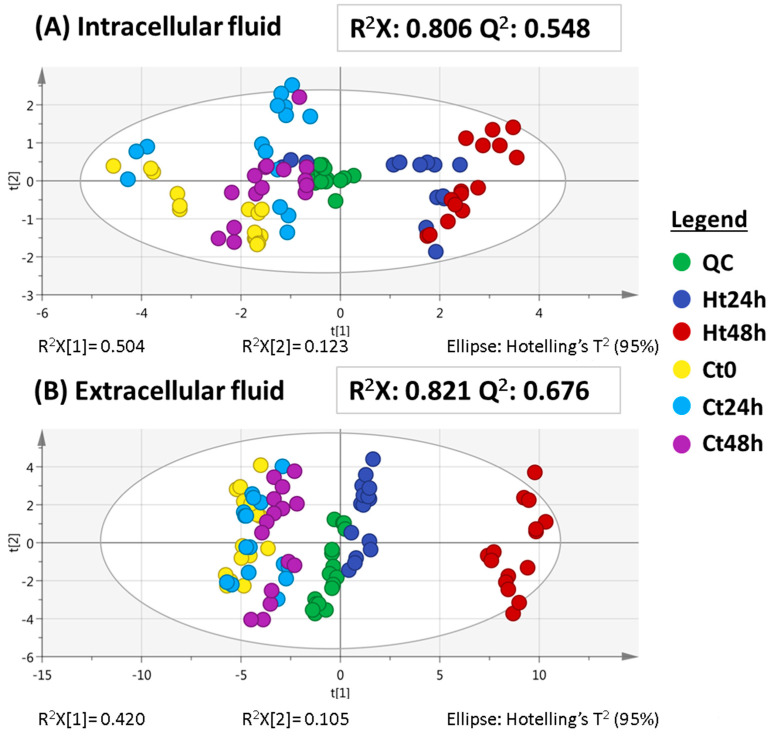
PCA for both intracellular fluid (**A**) and extracellular fluid (**B**) sequences at long times. Hotelling’s ellipses represent the 95% confidence limit. QC, quality control. Ht24h, hypoxia at 24 h. Ht48h, hypoxia at 48 h. Ct0, control at 0 h. Ct24h, control at 24 h. Ct48h, control at 48 h.

As can be seen in Figure 4, both the intracellular and extracellular fluids’ performance was successfully paying attention to the QC clustering. Note that, as in the first run (Figure 2), in the extracellular fluid at longer times (24 and 48 h), the experimental samples appeared to be better clustered and there was also a clear difference between the hypoxia groups and the control groups. Again, this supports the idea that there are more significant changes in the extracellular fluid than in the intracellular fluid. Interestingly, at longer times (Figure 4) in the extracellular fluid, there was a larger difference between the hypoxia groups (24 h vs. 48 h) when compared to the low hypoxia times from Figure 2, which means that longer durations of hypoxia might induce even more differences in the HK-2 metabolome.

PLS–DA models comparing Ct24h vs. Ht24h groups and Ct48h vs. Ht48h groups showed high R^2^X, R^2^Y and Q^2^ parameters for both fluids as can be seen in Table 3. CV-ANOVA and permutation tests proved that the models were perfectly valid (Figure S3).

Finally, all molecular features with a VIP value higher than 1.00 in Ht24h vs. Ct24h and Ht48h vs. Ct48h were selected to find those variables affected by hypoxia only at 24 h, only at 48 h, and at both times.

In the same way, 16 and 141 statistically significant features were retrieved from the intracellular and extracellular fluids and they are included in Table 4 and Table S2.

In the intracellular fluid, there were two significant variables at 24 h, six significant variables at 48 h, and eight significant variables were common at 24 and 48 h. In the same way as the previous sequences, the identification was carried out according to the MSI guidelines [19]. In this sense, the metabolites identified as level 1 were pyridoxine at 24 h and pyroglutamic acid and phenylalanine at 48 h.

In the extracellular fluid, there were 33 significant variables at 24 h, 53 significant variables at 48 h, and 55 significant variables were common at 24 and 48 h. In the intracellular fluid, pyridoxine at 24 h and pyroglutamic acid and phenylalanine at 48 h were identified as level 1. In the extracellular fluid, carnitine and valine at 24 h were also identified as level 1 whereas 4-hydroxy-3-methoxy-a-methylphenylalanine and thyronine at 48 h were identified as level 2. At both times, hippuric acid was identified as level 1 but pyroglutamic acid and N-(1-deoxy-1-fructosyl)phenylalanine were identified as level 2. Both 4-Hydroxy-3-methoxy-a-methylphenylalanine were identified in the same way as the sequences at short times. In this sense, N-(1-deoxy-1-fructosyl)phenylalanine could be identified because its MS/MS spectra matched the one reported in the literature [21].

It is important to highlight that in both intracellular and extracellular fluid, the number of altered metabolites is higher at 48 h, and additionally, in the extracellular fluid this number was even higher than in the intracellular fluid. This confirms once again our first hypothesis that longer hypoxia times induce larger metabolic differences in these cells and that most of the effects are seen in the extracellular fluid (difference in metabolites absorbed and/or excreted by the cells).

### 2.2. Evaluation of the Metabolic Effects Observed under Short and Long Times of Hypoxia

When the four metabolomic sequences are compared, certain aspects can be observed, which could show the relationship among them. In this sense, pyridoxine is common in the two sequences of intracellular fluid and, while the trend is upwards at short times, the trend is downwards at long times. Pyroglutamic acid is also common in the intracellular fluids and the trend is upwards in both cases but the trend of this amino acid derivate is downwards in the extracellular fluids at short times. On the other hand, L-4-hydroxy-3-methoxy-a-methylphenylalanine and thyronine are common in the two metabolomic sequences of extracellular fluids. Both metabolites are upwards regulated in these sequences. The trend of these metabolites is represented in Figures S4 and S5 and also the trend of the rest of the metabolites, which are only significant at short times or at long times, is represented in Figures S6 and S7. The biological relevance of these and the rest of the metabolites that were significantly altered will be described in the next section.

### 2.3. Biological Interpretation

Hypoxia is generally referred to as insufficient supply of oxygen and/or excessive oxygen consumption resulting in cellular stress because oxygen levels are below what is required for maintaining normal cellular function [5]. Renal hypoxia is a relevant factor in the pathophysiology of both acute kidney injury and chronic kidney disease, proximal tubules being the most vulnerable part of the renal tubules in the face of hypoxia [22]. This is because of the high oxygen consumption by proximal tubular cells as a consequence of their energy-dependent activities of reabsorption [23]. To preserve tissue function under hypoxia, proximal tubular cells undergo alterations in their metabolism, although the impact of this metabolic re-wiring on most cellular metabolic pathways is far from being fully elucidated. The current study has identified several potentially relevant metabolic changes induced by hypoxia in the intracellular or extracellular fluids of HK-2 cells, which are discussed below.

Plasma levels of acetylcarnitine, butyrylcarnitine and carnitine have been associated with higher odds of chronic kidney disease [24]. In our work, the intracellular content in acetylcarnitine (Figure S6A)—an ester involved in energy storage—decreased during short-term hypoxia (i.e., 0.5 or 5 h hypoxia). Acetylcarnitine can be enzymatically cleaved in the mitochondria so that it renders carnitine and acetyl CoA, which can be used as an energy source [25]. Since proximal tubular cells are completely dependent on aerobic respiration for ATP production [26], we propose that the reduced intracellular levels of acetylcarnitine are the consequence of its use for the metabolic needs of proximal tubular cells during short-term hypoxia. The intracellular content of butyrilycarnitine also decreased after 0.5 h of hypoxia but increased 5 h later (Figure S6B), these changes being likely related to alterations in the branched chain amino acids catabolism because short-chain acylcarnitines are products of these enzymatic reactions [27]. Finally, we found carnitine in the extracellular medium of HK-2 cells cultured under control conditions, and the exposure of cells to long-term hypoxia (24/48 h) resulted in lower extracellular levels of carnitine (Figure S7C). Since the culture medium does not contain carnitine, it is most likely that its presence in the extracellular fluid is due to its export from the intracellular fluid. Although carnitine is subjected to extensive renal tubular reabsorption [28] (more than 95% of the carnitine filtered in the kidney is reabsorbed by the proximal tubules), our results in the control proximal tubular HK-2 cells indicate that they are also able to secrete carnitine to the extracellular medium, which suggests the presence of a carnitine export transporter in HK-2 cells. In this context, we speculate that the inhibition of this transporter by long-term hypoxia would explain the lower levels of carnitine found in the extracellular medium of HK-2 cells under hypoxia. It remains to be elucidated whether proximal tubular cells also secrete carnitine in vivo, thereby contributing to the urinary levels of carnitine.

Pyridoxine is one of the vitamin B6 vitamers and it is included in the formulation of cell culture media because mammalian cells are unable to synthesize it. Proximal tubular cells take up pyridoxine from the culture medium via carrier mediated mechanisms [29,30,31] and transform it into its active form, pyridoxal 5′-phosphate [32]. Pyridoxal 5′-phosphate is utilized as a coenzyme in lipid, amino acid, protein, and carbohydrate metabolism [33] so it is not surprising that the changes induced by hypoxia in metabolic pathways affect the content of pyridoxine in HK-2 cells (Figure S4B). The increase in intracellular pyridoxine after short-term hypoxia probably reflects a decreased activity of those metabolic reactions which require pyridoxal 5′-phosphate. On the contrary, long-term hypoxia resulted in diminished intracellular levels of pyridoxine, which might be due [34], at least in part, to reduced ATP generation: hypoxia inhibits mitochondrial oxidative phosphorylation (an oxygen-dependent process) and compromises ATP generation because it now depends more on glycolysis, which generates much less ATP [35]. Due to the fact that pyridoxine uptake in HK-2 cells is an energy-dependent process [31], we hypothesize that it may be diminished in HK-2 cells under long-term hypoxia as a consequence of the reduced ATP generation. This would contribute to their lower intracellular content in pyridoxine. An additional contribution might depend on the consumption of cell pyridoxine in hypoxia activated metabolic pathways such as glycolysis, glycogenolysis, or amino acid metabolism, because pyridoxal 5′-phosphate-dependent enzymes play essential roles in them [34]. Of course, further studies are needed to establish the mechanism responsible for the lower intracellular content in pyridoxine found in HK-2 cells under hypoxia.

Thyronine levels in the extracellular fluid of HK-2 cells were increased after both short- and long-term hypoxia. Thyronine, which is the thyroxine nucleus devoid of its four iodine atoms, has been identified in human urine [36]. On the other hand, the converting activity of thyroxine to 3,3′,5-triiodothyronine—which exerts the majority of thyroid hormone action—has been reported in proximal tubular cells [37]. Given that thyroxine is a normal constituent of serum, we hypothesize that an increase in its total deiodination by HK-2 cells, followed by secretion to the medium of the deiodinated product (i.e., thyronine), may play a role in the enhanced presence of thyronine in the extracellular fluid.

For thyronine and L-4-hydroxy-3-methoxy-a-methylphenylalanine in extracellular fluid, their level increased after short hypoxia times then came to a similar level to that of normal cells after 24 h of hypoxia, and finally increased again after 48 h of hypoxia. In other words, hypoxia induced a time-dependent, inverted U-shaped effect on the extracellular levels of both metabolites. There is not any obvious explanation for these results. However, one may speculate biphasic changes in the activity of unidentified, hypothetical transporters responsible for the export of these metabolites and/or in the activity of non-well known enzymes involved in their metabolism, but the clarification of the true underlying molecular mechanisms awaits further research. Non-linear relationships (either U-shaped or inverted U-shaped) between dose or time and biological effects are not unusual in biological studies: we found more than 9000 articles in a search in PubMed using the term “U-shaped[Title/Abstract]”. However, the complexity of the mechanisms on which these non-linear associations are based makes difficult their full elucidation. In fact, in many cases, researchers just describe them and do not even attempt any interpretation.

Several metabolomics studies have associated phenylacetylglycine—a minor product of mitochondrial fatty acid oxidation—with renal damage [38,39,40]. Increased urinary excretion of phenylacetylglycine has been related to mitochondrial fatty acid oxidation [41]. In the current study, we found that hypoxia (for 0.5 or 5 h) increased phenylacetylglycine levels in the culture medium (Figure S7A). Although this suggests that lipid metabolism may be affected by short-term hypoxia in proximal tubular cells, this hypothesis should be experimentally confirmed.

The main changes in the amino acids present in the extracellular medium of proximal tubular cells under hypoxia (Figures S5 and S7) involved: (i) branched chain amino acids and a number of their derivatives such as valine, N-(1-deoxy-1-fructosyl)leucine and N-(1-deoxy-1-fructosyl)isoleucine; (ii) derivatives from aromatic amino acid phenylalanine such as N-(1-deoxy-1-fructosyl)phenylalanine and L-4-hydroxy-3-methoxy-a-methylphenylalanine; and (iii) pyroglutamic acid and hippuric acid, which derive from cysteine and glycine, respectively. N-1-Deoxy-1-fructosyl derivatives are Amadori products from the Maillard reaction, which are formed by non-enzymatic glycation of amino acids by reducing sugars [42]. The review of the literature does not provide any insight into the biological meaning of the increase in N-1-deoxy-1-fructosyl amino acid derivatives in the culture medium from hypoxia-exposed HK-2 cells and the same is true for L-4-hydroxy-3-methoxy-a-methylphenylalanine. We can only affirm that these amino acid derivatives come from HK-2 cells because they are not present in the culture medium formulation. Regarding the decrease in valine in the extracellular fluid, which is a component of the culture medium, it is most likely due to its enhanced uptake from the culture medium by HK-2 cells subjected to long-term hypoxia: previous work in rabbits has shown that proximal tubular cells take up valine in an Na^+^-dependent, H^+^-independent and electrogenic manner [43]. Since gluconeogenic amino acids are the main precursor of glucose synthesis in the gluconeogenesis pathway, we speculate that the decrease in valine in the extracellular fluid is the consequence of its selective uptake for use in gluconeogenesis in an attempt to replenish glucose consumed by HK-2 cells during long-term hypoxia-activated glycolysis. It is worth mentioning that the intracellular content of another gluconeogenic amino acid, phenylalanine, increased in long-term hypoxia, which reinforces the view of the selective use of valine by HK-2 cells for gluconeogenic purposes. Pyroglutamate (5-oxoproline) content in the intracellular fluids of HK-2 cells subjected to both short- and long-term hypoxia were also increased. In mammalian cells, pyroglutamate is an intermediate in glutathione synthesis but it is also a degradation product of glutathione, a major source of antioxidants [44], and this is why pyroglutamate accumulates during oxidative stress [45,46]. Reactive oxygen species-dependent oxidative stress may be induced by hypoxia [47] and, therefore, one may speculate that the accumulation of pyroglutamate in HK-2 cells subjected to long-term hypoxia is due to the degradation of glutathione caused by oxidative stress. Interestingly, pyroglutamate itself has been shown to induce oxidative stress [45,46]. Long-term hypoxia also determined a decrease in the pyroglutamate levels in the extracellular fluid, which was most likely due to its diminished release from HK-2 cells, given that pyroglutamate is not present in the culture medium formulation. Finally, the increase in hippuric acid in the extracellular medium of HK-2 cells under long-term hypoxia may be also pathologically relevant. Hippuric acid is an acyl glycine formed in the glycine conjugation pathway of benzoate in the mitochondrial matrix of proximal tubular cells and then secreted to the urine [48,49,50]. Extracellular hippuric acid exhibits oxidative stress-associated toxicity in endothelial cells [51,52]. Whether hippuric acid also induces oxidative stress in proximal tubular cells remains to be investigated.

## 3. Materials and Methods

### 3.1. Reagents and Solvents

The solvents and reagents employed in this work were of analytical grade or higher. Acetonitrile, formic acid and methanol were from Thermo Fisher Scientific (Madrid, Spain). The water used to make solutions was obtained through a Milli-Q System (Millipore, Bedford, MA, USA).

The standards required for verifying the metabolite identification were: acetylcarnitine, allylcysteine, butyrylcarnitine, carnitine, cystine, dopamine, glycine, hippuric acid, 5-hydroxydopamine, 2-methylhippuric acid, 3-methylhippuric acid, methylpyrrolidine, norepinephrine, norvaline, phenylacetylglycine, phenylalanine, pyridoxine, pyroglutamic acid, sphinganine, and valine. They were all acquired from Sigma Aldrich (Madrid, Spain).

### 3.2. HK-2 Cell Line Culture

Human proximal tubular HK-2 cells were from the American Type Culture Collection (ATCC, Rockville, MD, USA). The culture was maintained in 5% CO_2_ at 37 °C in DMEM/F12 supplemented with 1% penicillin/streptomycin/amphotericin B, 10% fetal bovine serum (FBS), 1% insulin-transferrin-selenium (ThermoFisher. Grand Island, NY, USA) and 1% glutamine (Invitrogen. Carlsbad, CA, USA). The metabolomics study was carried out in P35 culture dishes cells (5 × 10^5^ cells/mL)

Cells were plated at 90% confluence and, when they were completely attached, they were cultured under hypoxic conditions (1% oxygen) at different times (0.5, 5, 24, or 48 h) or control conditions (21% oxygen) and were kept for as long as the ones at hypoxia for control purposes. The in vivo 200 hypoxia workstation (Ruskinn Technology, West Yorkshire, UK) was used to perform hypoxia experiments. For each treatment, six replicates were used. Five of them for metabolomic study and the remaining replicate for cell counting and protein quantification.

A hemocytometer, which allows the discrimination between viable and dead cells using trypan blue exclusion, was employed for measuring manually the cell number whereas the Pierce BCA-200 Protein Assay Kit (Thermo Fisher, Grand Island, NY, USA) was used for measuring the protein content in each well, according to the instructions provided by the manufacturer (Thermo Fisher, Grand Island, NY, USA). The cell number and the protein content are summarized in Tables S3 and S4.

Regarding the exometabolome analysis, the extracellular media was collected and kept at −80 °C until further processing. Then, 50 mM PBS (pH 7.4) was used to wash cells. Cells were trypsinized and then resuspended in 1 mL of DMEM/F12. Regarding the endometabolome analysis, cells were centrifuged for 5 min at 2500 rpm and cell pellets were stored at −80 °C until further use.

### 3.3. Protein Isolation and Western-Blotting

Adherent cells were plated into six-well plates (3 × 10^5^ cells/well). After treatments, the dishes containing the cells were placed on ice and washed with ice-cold PBS. PBS was discarded by aspiration and then the ice-cold lysis buffer (Promega, Madison, WI, USA), with a cocktail of protease inhibitors (Roche, Basel, Switzerland), was added and the cells were scraped. Cells were maintained for 30 min on ice, then the cells suspension was transferred into a microcentrifuge tube and was centrifuged for 5 min at 4000× *g*. The lysate was prepared to determine the protein concentration using the Bradford assay. Approximately 30 μg of total protein was loaded in 8% SDS–polyacrylamide gel. After electrophoresis, proteins from the gel were transferred to a PVDF membrane using transfer buffer 1× (380 mM glycine, 50 mM Tris-HCl and 20% methanol). After 1 h, mouse anti-HIF1α antibodies (working dilution 1:1000) and anti-tubulin antibody (1:5000), as a control, were used to incubate blots at 4 °C. After 24 h, blots were incubated with the corresponding secondary antiserum (1:4000). Signals were detected with enhanced chemiluminescence reagent (Amersham Healthcare, Buckinghamshire, UK).

### 3.4. Optimized Sample Preparation Protocol

The procedure included in this section was previously optimized by our research group [15]. To carry out the extraction of the intracellular metabolites, cell pellets from Section 3.2 were extracted with 400 µL of 75% (*v*/*v*) MeOH in water. Then, the mixture was vortexed for 30 s, left in an ice bath for 5 min, and submitted to centrifugation (14,000× *g* for 5 min at 4 °C). Once the supernatant was collected, it was separated into two parts of 200 µL each. Later, these fractions were concentrated for 3.5 h until dryness and 100 µL of water was added to reconstitute them. The solutions were vortexed for 30 s and centrifuged at 14,000× *g* for 5 min at 4 °C and placed into inserts for further analysis.

To carry out the extraction of the extracellular metabolites, 300 µL MeOH was added to 100 µL of extracellular fluid from Section 3.2. Then, the mixture was vortexed for 30 s, left in an ice bath for 5 min, and submitted to centrifugation (14,000× *g* for 5 min at 4 °C). In a similar way to the intracellular fluid, the supernatant collected was separated into two fractions (200 µL each) and evaporated for 3.5 h until dryness. The reconstitution step was the same as for the extraction of intracellular metabolites.

An aliquot of each sample was pooled in order to obtain the QC samples of the metabolomic sequences.

### 3.5. Liquid Chromatography–Mass Spectrometry Analysis

The LC system used was an 1100 series HPLC (Agilent Technologies, Germany) coupled to a 6530 series quadrupole time-of-flight (QTOF) mass spectrometer (Agilent Technologies, Germany) employing a Jet Stream orthogonal electrospray ionization (ESI). For MS control and data acquisition, Agilent Mass Hunter Qualitative Analysis software (B.07.00) was employed. To carry out the mass correction for the positive ionization mode, a solution containing two reference ions (m/z 121.0509 (C_5_H_4_N_4_) and m/z 922.0098 (C_18_H_18_O_6_N_3_P_3_F_24_) was continuously infused at 15 µL/min into the system through a 25 mL Gastight 1000 Series Hamilton syringe (Hamilton Robotics, Bonaduz, Switzerland) on a NE-3000 pump (New Era Pump Systems Inc., Farmingdale, NY, USA).

A C_18_ Ascentis Express column (Sigma, St Louis, MO, USA), having dimensions of 100 × 2.1 mm i.d. (fused-core^®^ particles with 0.5 µm thick, porous shell and an overall particle size of 2.7 µm). A guard column (5 × 2.1 mm i.d.) of the same composition was used. During the sample analysis, the column was kept at 40 °C, and an injection volume of 10 µL and a flow rate of 0.4 mL/min were employed.

Water (eluent A) and acetonitrile (eluent B), both with 0.1% formic acid were used as mobile phases. The gradient used consisted of: 5% B to 100% B in 30 min, 100% B for 5 min, returning to starting conditions (5% B) in 1 min, and holding it for 15 min.

The ionization source conditions were: a capillary voltage of 3000 V with a nozzle voltage of 0 V; nebulizer pressure at 35 psig; sheath gas of jet stream of 6.5 L/min at 275 °C; and drying gas of 10 L/min at 275 °C. The fragmentator (cone voltage after capillary) was set at 100 V and the skimmer and octapole voltage was at 750 V. MS analyses were performed in the positive ESI mode, with mass range set at m/z 70–1600 (extended dynamic range) in full scan resolution mode at a scan rate of 2 scans per second.

To carry out the metabolite identification by MS/MS analyses, the [M + H]^+^ ions of the metabolites were selected as precursor ions at the desired retention time with a collision energy of 20 V. In all cases where the voltage applied was not enough to fragment the precursor ion, it was set at 30 V.

### 3.6. Metabolomic Sequence

To start the metabolomic sequence, first, several blanks and QCs samples were analyzed in order to assure the repeatability in the system. As a common practice in metabolomics, samples were randomized and for every five samples analyzed, a QC was injected. Finally, the sequences were finalized with the analysis of several QC samples.

### 3.7. Data Treatment and Analysis

The Molecular Feature Extraction tool in Mass Hunter Qualitative Analysis (B.07.00) was employed to find molecular features, taking into account the adducts in the positive ionization mode (H^+^, Na^+^, K^+^, and NH_4_^+^). A minimum of 12,000 counts (calculated as three times the signal-to-noise (S/N) ratio) was used for the extraction of the molecular features.

Data were filtered and aligned using the Agilent Mass Profiler Professional tool (B.02.00). Retention time data were 0.1% with a window of 0.15 min. Mass tolerance was 20.0 ppm with a mass window of 2.0 mDa.

The multivariate statistical analysis was carried out employing SIMCA 14.0 (Umetrics, Umeå, Sweden). Prior to PCA and PLS–DA, it was necessary to carry out log-transformation, Pareto scaling, and normalization against the cell number (see Section 3.2). To select significant molecular features, variable importance in the projection (VIP) values of the first component of the PLS–DA models were employed.

To carry out the box plots and univariate statistical analysis (Mann Whitney U test), R (http://www.R-project.org) was used.

### 3.8. Identification of Metabolites

Those molecular features which showed the most important differences in the PLS–DA models were selected for the identification stage. The CEU Mass Mediator database from the Centre for Metabolomics and Bioanalysis (CEMBIO, Spain) [53] was employed for the identification, taking into account the obtained accurate mass values, assuming an error of 30 ppm. This database includes several databases, such as KEGG (https://www.genome.jp/kegg/), HMDB (http://www.hmdb.ca/), METLIN (https://metlin.scripps.edu), and LipidMaps (http://www.lipidmaps.org/), and allows the simultaneous search of metabolites (accessed on April 2021). The possible metabolites were filtered based on the probability of finding them in biological samples and not considering exogenous compounds such as drugs or compounds of plant origin.

To carry out the identification of those important molecular features, different levels were established according to the MSI guidelines [19]. In this sense, those metabolites identified unequivocally matching retention time, accurate monoisotopic mass, and MS/MS fragmentation patterns of that of the standard solution (see Section 3.1), were classified as level 1. The metabolites for which identification was based on accurate mass and fragmentation, compared to available reference databases, such as the HMDB database (http://www.hmdb.ca/), CFM-ID (cfmid.wishartlab.com) (both accessed on April 2021) and the literature, were classified as level 2. Finally, those metabolites whose accurate mass was known but for which no precise metabolites could be annotated, were classified as level 4.

## 4. Conclusions

In this work, the metabolic differences between HK-2 cells at different hypoxic times (0.5, 5, 24 and 48 h) have been evaluated for the first time. An untargeted metabolomics approach was chosen on the basis of exploring the changes observed in metabolism without a prior targeted pathway in mind. We used LC–MS to assess both the intracellular and extracellular fluids in order to obtain a broad metabolite coverage and to evaluate which metabolites were taken up and/or released by these cells. The results indicate that, under hypoxic conditions, cells undergo metabolic re-wiring and it can be observed that, as the duration of hypoxia increases, the number of molecular features increases, especially in the extracellular fluid; namely, alterations in the catabolism of amino acids, including aromatic, branched-chain amino acids and derivatives such as Amadori products, as well as changes in pyridoxine metabolism, mitochondrial fatty acid oxidation and others. However, these so far remain merely as a hypothesis that must be validated in vivo.

## Figures and Tables

**Table 1:** PLS–DA pairwise models parameters (R^2^X, R^2^Y, and Q^2^) and the F and *p*-values of the cross-validated ANOVA (CV-ANOVA) for the two analytical sequences at 0.5 and 5 h.

PLS-DA Models	R^2^X	R^2^Y	Q^2^	CV-ANOVA	
**Intracellular fluid**					
Ht0.5h vs. Ct0.5h	0.620	0.801	0.732	F(17.1)	*p*(7.3 × 10^−7^)
Ht5h vs. Ct5h	0.794	0.982	0.968	F(102.8)	*p*(1.9 × 10^−15^)
**Extracellular fluid**					
Ht0.5h vs. Ct0.5h	0.606	0.998	0.986	F(186.7)	*p*(1.2 × 10^−16^)
Ht5h vs. Ct5h	0.652	0.996	0.978	F(117.0)	*p*(1.5 × 10^−15^)

**Table 2:** Statistically significant metabolites found in the intracellular and extracellular fluid analysis at 0.5 and 5 h.

#	RT (min)	Molecular Formula	Metabolite	Identification Level *	Monoisotopic Mass (Da)	Mass Error (ppm)	Main Fragments (MS/MS)	VIP (Trend) **	
								Ht0.5h vs. Ct0.5h	Ht5h vs. Ct5h
**Intracellular fluid**									
**Significant metabolites at 5 h**									
1	0.8	C_5_H_7_NO_3_	Pyroglutamic acid	1	129.0424	1.5	56.0516, 84.0469	0.81 (↑)	1.19 (↑)
2	1.8	C_11_H_21_NO_4_	Butyrylcarnitine	1	231.1485	6.1	85.0283	0.01 (↓)	1.51 (↑)
**Significant metabolites at 0.5 and 5 h**									
3	0.8	C_9_H_17_NO_4_	Acetylcarnitine	1	203.1188	14.8	85.0295	2.20 (↓)	2.13 (↓)
4	0.8	C_8_H_11_NO_3_	Pyridoxine	1	169.0765	15.4	134.0593, 152.0705	1.46 (↑)	1.39 (↑)
**Extracellular fluid**									
**Significant metabolites at 0.5 h**									
5	5.4	C_10_H_11_NO_3_	Phenylacetylglycine	1	193.0763	12.4	135.0445, 107.0516	1.45 (↑)	0.59 (↑)
**Significant metabolites at 5 h**									
6	0.9	C_12_H_23_NO_7_	N-(1-Deoxy-1-fructosyl)leucineor N-(1-deoxy-1-fructosyl)isoleucine	2	293.1525	17.1	230.1378, 258.1333, 276.1420	0.35 (↑)	1.12 (↑)
**Significant metabolites at 0.5 and 5 h**									
7	3.7	C_11_H_15_NO_4_	L-4-Hydroxy-3-methoxy-a-methylphenylalanine	2	225.1011	4.4	180.1008	1.85 (↑)	1.46 (↑)
8	6.4	C_15_H_15_NO_4_	Thyronine	2	273.1013	4.4	228.1009	2.08 (↑)	1.56 (↑)

* Identification according to the MSI guidelines [19]: level 1 (unequivocal identification matching retention time, accurate mass and MS/MS of that of the standard solution); level 2 (identification based on accurate mass, and fragmentation, compared to available reference database). ** ↑: The metabolite (on average) is more abundant in the hypoxia condition; ↓: The metabolite (on average) is less abundant in the hypo*x*ia condition.

**Table 3:** PLS–DA pairwise models’ parameters (R^2^X, R^2^Y, and Q^2^) and the F and *p*-values of the cross-validated ANOVA (CV-ANOVA) for the two analytical sequences 24 and 48 h.

PLS-DA models	R^2^X	R^2^Y	Q^2^	CV-ANOVA	
**Intracellular fluid**					
Ht24h vs. Ct24h	0.616	0.923	0.873	F(39.9)	*p*(1.6 × 10^−10^)
Ht48h vs. Ct48h	0.649	0.948	0.935	F(98.8)	*p*(6.1 × 10^−15^)
**Extracellular fluid**					
Ht24h vs. Ct24h	0.493	0.985	0.967	F(171.3)	*p*(2.8 × 10^−18^)
Ht48h vs. Ct48h	0.572	0.984	0.983	F(776.8)	*p*(1.4 × 10^−24^)

**Table 4:** Statistically significant metabolites found in the intracellular and extracellular fluid analysis at 24 and 48 h.

#	RT (min)	Molecular Formula	Metabolite	Identification Level *	Monoisotopic Mass (Da)	Mass Error (ppm)	Main Fragments (MS/MS)	VIP (Trend) **	
								Ht24h vs. Ct24h	Ht48h vs. Ct48h
**Intracellular fluid**									
**Significant metabolites at 24 h**									
1	0.8	C_8_H_11_NO_3_	Pyridoxine	1	169.0746	4.1	134.0597, 152.0691	1.16 (↓)	0.80 (↓)
**Significant metabolites at 48 h**									
2	0.8	C_5_H_7_NO_3_	Pyroglutamic acid	1	129.0437	8.5	84.0445, 56.0491	0.98 (↑)	1.25 (↑)
3	1.3	C_9_H_11_NO_2_	Phenylalanine	1	165.0781	5.5	120.0801, 103.0534	0.40 (↑)	1.07 (↑)
**Extracellular fluid**									
**Significant metabolites at 24 h**									
4	0.7	C_7_H_15_NO_3_	Carnitine	1	161.1010	5.5	85.0260, 103.0327, 60.0790	1.16 (↓)	0.83 (↓)
5	0.8	C_5_H_11_NO_2_	Valine	1	117.0770	16.2	72.0815, 55.0549	1.16 (↓)	0.71 (↓)
**Significant metabolites at 48 h**									
6	3.3	C_11_H_15_NO_4_	L-4-Hydroxy-3-methoxy-a-methylphenylalanine	2	225.0996	2.2	180.1029	0.07 (↑)	1.13 (↑)
7	6.2	C_15_H_15_NO_4_	Thyronine	2	273.0997	1.5	228.1030	0.51 (↑)	1.51 (↑)
**Significant metabolites at 24 and 48 h**									
8	0.8	C_5_H_7_NO_3_	Pyroglutamic acid	2	129.0431	3.9	84.0416,56.0488	1.28 (↓)	1.47 (↓)
9	1.3	C_15_H_21_NO_7_	N-(1-deoxy-1-fructosyl)phenylalanine	2	327.1295	7.0	310.1271, 292.1159, 166.0846, 178.0845	1.27 (↑)	1.21 (↑)
10	4.0	C_9_H_9_NO_3_	Hippuric acid	1	179.0580	1.1	105.0327, 77.0390	1.27 (↑)	1.13 (↑)

* Identification according to the MSI guidelines [19]: level 1 (unequivocal identification matching retention time, accurate mass and MS/MS of that of the standard solution); level 2 (identification based on accurate mass, and fragmentation, compared to available reference database). ** ↑: The metabolite (on average) is more abundant in hypoxia condition; ↓: The metabolite (on average) is less abundant in hypoxia condition.

## Data Availability

Datasets of the four metabolomic sequences are available with the manuscript.

## Supplementary Materials

The following are available online at https://www.mdpi.com/article/10.3390/ijms22147399/s1.
