# Supplementary material for: Exploratory Metabolomic Analysis Based on Reversed-Phase Liquid Chromatography–Mass Spectrometry to Study an In Vitro Model of Hypoxia-Induced Metabolic Alterations in HK-2 Cells
Source: Int J Mol Sci. 2021 Jul 9;22(14):7399. doi: 10.3390/ijms22147399 (PMC8304667; doi:10.3390/ijms22147399)
Supplement: Supplementary file 1 [file ijms-22-07399-s001.zip › ijms-1274485-supplementary.pdf]

Exploratory metabolomic analysis based on reverse phase liquid chromatography-mass spectrometry to study an *in vitro* model of hypoxia-induced metabolic alterations in HK-2 cells

Samuel Bernardo-Bermejo[1], Elena Sánchez-López[2#], Lei Tan[1,3], Selma Benito-Martínez[4,5], Zhengjin Jiang[3], María Castro-Puyana[1,6], Francisco Javier Lucio Cazaña[4], María Luisa Marina[1,6*]

[1]Universidad de Alcalá. Departamento de Química Analítica, Química Física e Ingeniería Química. Ctra. Madrid-Barcelona Km. 33.600, 28871, Alcalá de Henares (Madrid), Spain.

[2]Department of Human Genetics, Leiden University Medical Center, Leiden, the Netherlands.

[3]Institute of Pharmaceutical Analysis, College of Pharmacy, Jinan University, Guangzhou, 510632, China.

[4]Universidad de Alcalá. Departamento de Biología de Sistemas, Ctra. Madrid-Barcelona Km. 33.600, 28871 Alcalá de Henares (Madrid), Spain.

[5]Universidad de Alcalá. Ramón y Cajal" Health Research Institute (IRYCIS), Madrid, Spain.

[6]Universidad de Alcalá. Instituto de Investigación Química Andrés M. del Río. Ctra. Madrid-Barcelona Km. 33.600, 28871, Alcalá de Henares (Madrid), Spain.

Current affiliation: Center for Proteomics and Metabolomics, Leiden University Medical Center, Leiden, the Netherlands.

***Correspondence:** Universidad de Alcalá. Departamento de Química Analítica, Química Física e Ingeniería Química. Ctra. Madrid-Barcelona Km. 33.600, 28871, Alcalá de Henares, Madrid, España.

E-mail: mluisa.marina@uah.es

Fax: +34-91 885 4971

Tel: +34-91 885 4935

Figures

Figure S1. Permutation tests of PLS-DA models at short times.

Figure S2. Total ion chromatograms of four samples of the two metabolomic sequences at long times: A) Control sample at 48 h of intracellular fluid, B) Hypoxia sample at 48 h of intracellular fluid, C) Control sample at 48 h of extracellular fluid, D) Hypoxia sample at 48 h of extracellular fluid. Experimental conditions detailed on *section 3.5*.

Figure S3. Permutation tests of PLS-DA models at long times.

Figure S4. Box-plots of the common metabolites identified in the intracellular fluid at short and long times. The p-value of Mann Whitney U test is included.

Figure S5. Box-plots of the common metabolites identified in the extracellular fluid at short and long times. The p-value of Mann Whitney U test is included.

Figure S6. Box-plots of the metabolites identified in the intracellular fluid at short or long times. The p-value of Mann Whitney U test is included.

Figure S7. Box-plots of the common metabolites identified in the extracellular fluid at short and long times. The p-value of Mann Whitney U test is included.

Figure S1

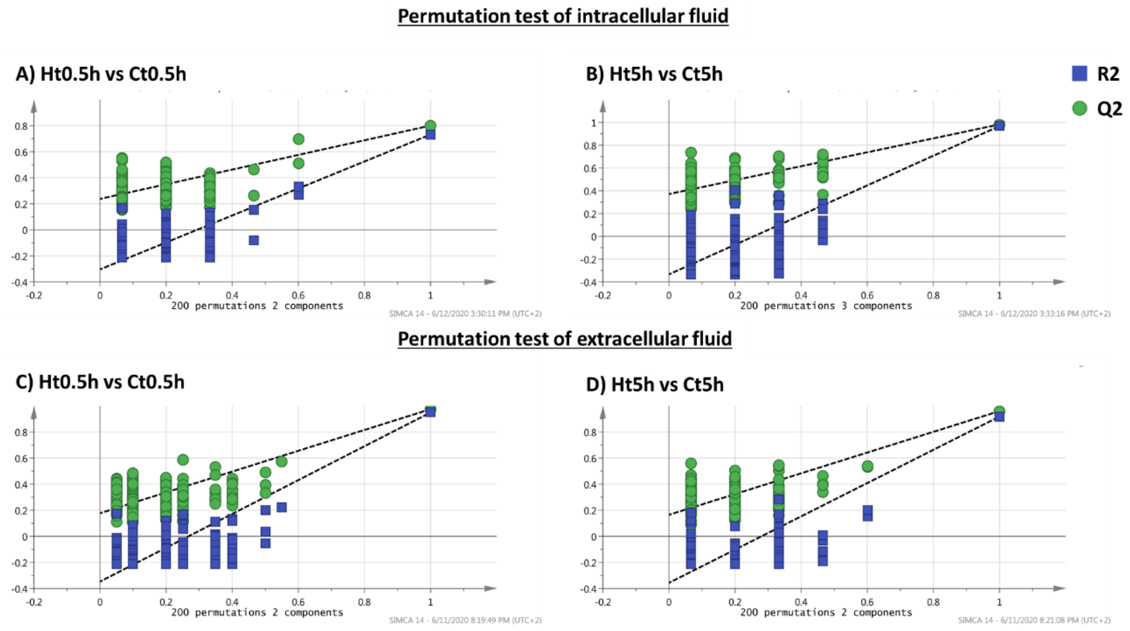
Permutation test of intracellular fluid
A) Ht0.5h vs Ct0.5h
B) Ht5h vs Ct5h
R2
Q2
200 permutations 2 components
200 permutations 3 components
Permutation test of extracellular fluid
C) Ht0.5h vs Ct0.5h
D) Ht5h vs Ct5h
200 permutations 2 components
200 permutations 2 components

Figue S2.

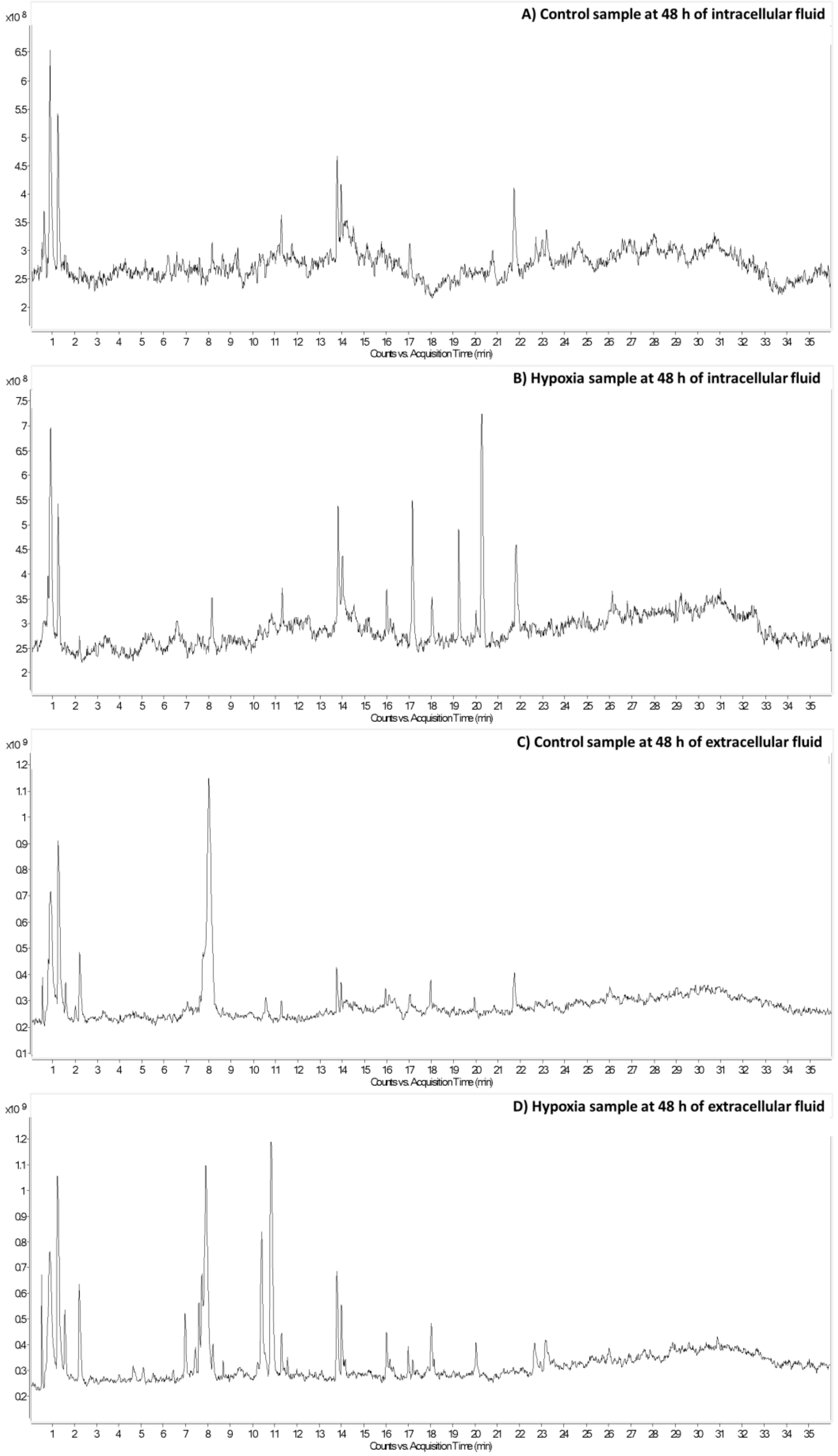
A) Control sample at 48 h of intracellular fluid
x10 8
Counts vs. Acquisition Time (min)
B) Hypoxia sample at 48 h of intracellular fluid
x10 8
Counts vs. Acquisition Time (min)
C) Control sample at 48 h of extracellular fluid
x10 9
Counts vs. Acquisition Time (min)
D) Hypoxia sample at 48 h of extracellular fluid
x10 9
Counts vs. Acquisition Time (min)

Figure S3

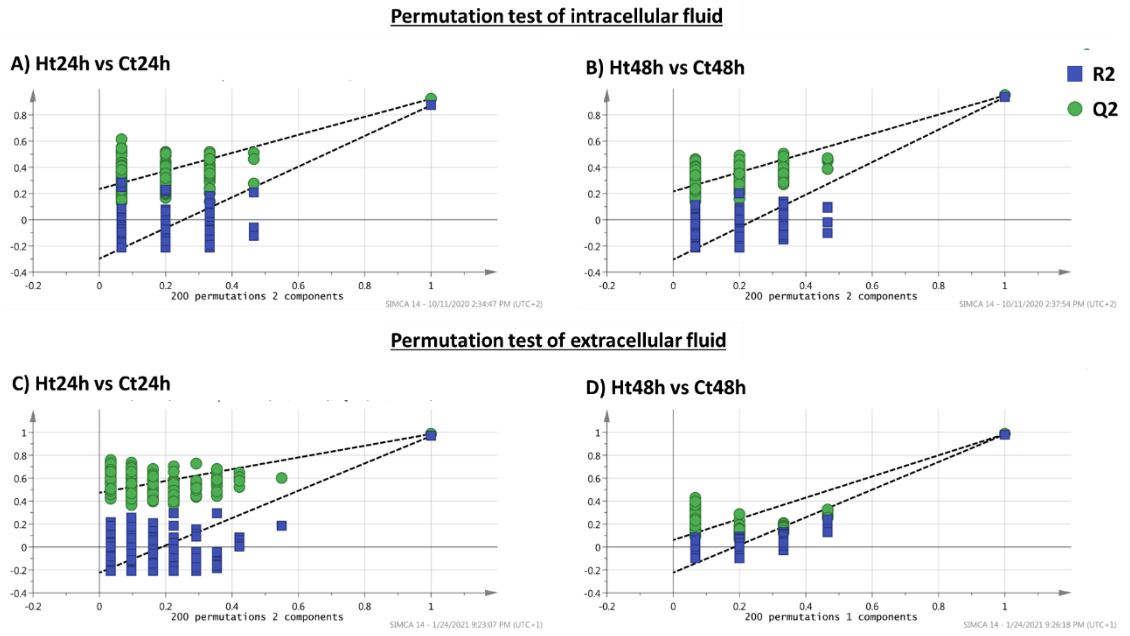
Permutation test of intracellular fluid
A) Ht24h vs Ct24h
200 permutations 2 components
SIMCA 14 - 10/11/2020 2:34:47 PM (UTC+2)
B) Ht48h vs Ct48h
R2
Q2
200 permutations 2 components
SIMCA 14 - 10/11/2020 2:37:54 PM (UTC+2)
Permutation test of extracellular fluid
C) Ht24h vs Ct24h
200 permutations 2 components
SIMCA 14 - 1/24/2021 9:23:07 PM (UTC+1)
D) Ht48h vs Ct48h
200 permutations 1 components
SIMCA 14 - 1/24/2021 9:26:18 PM (UTC+1)

Figure S4

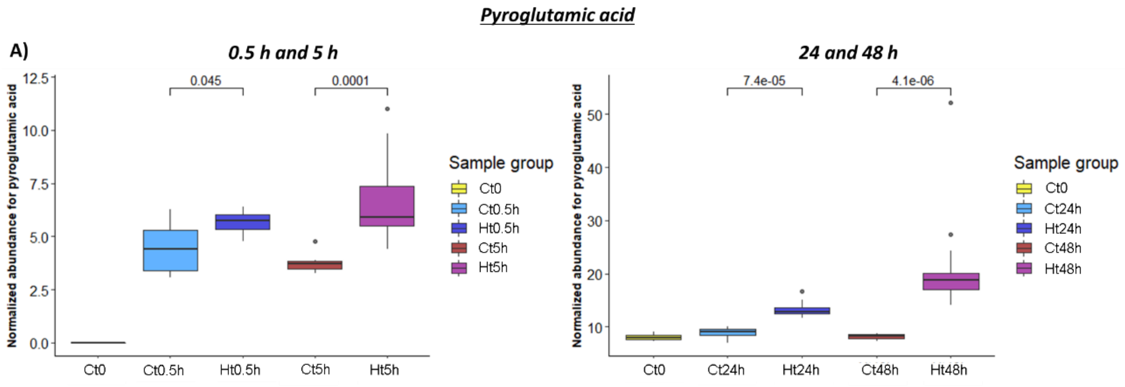

Pyroglutamic acid
A)
0.5 h and 5 h
Normalized abundance for pyroglutamic acid
0.045
0.0001
Ct0
Ct0.5h
Ht0.5h
Ct5h
Ht5h
Sample group
24 and 48 h
7.4e-05
4.1e-06
Ct24h
Ht24h
Ct48h
Ht48h

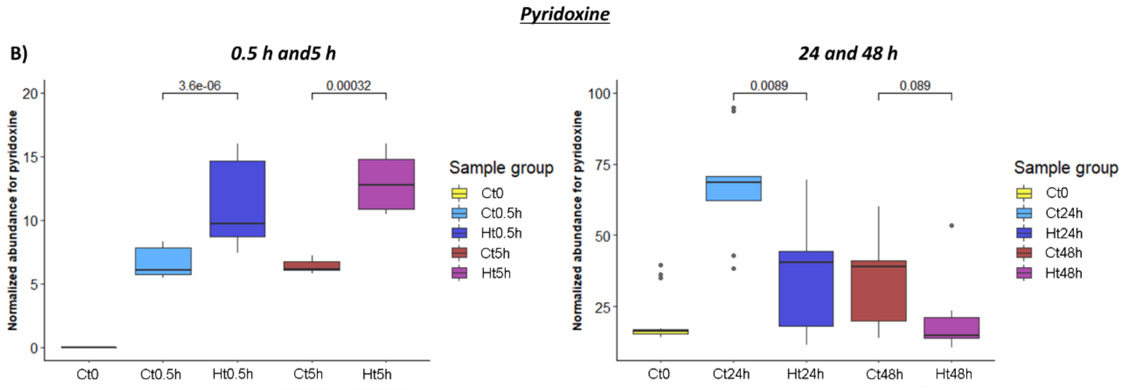

Pyridoxine
B)
0.5 h and5 h
Normalized abundance for pyridoxine
3.6e-06
0.00032
Ct0
Ct0.5h
Ht0.5h
Ct5h
Ht5h
Sample group
24 and 48 h
0.0089
0.089
Ct24h
Ht24h
Ct48h
Ht48h

Figure S5

L-4-Hydroxy-3-methoxy-a-methylphenylalanine

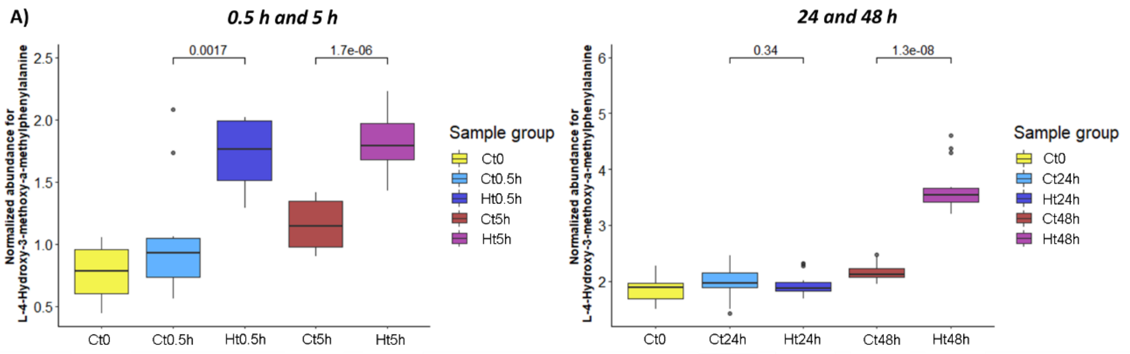

Thyronine

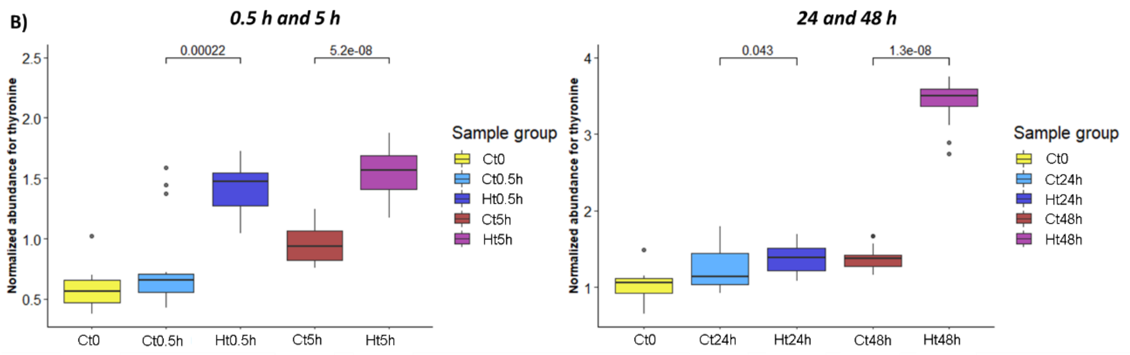

Figure S6

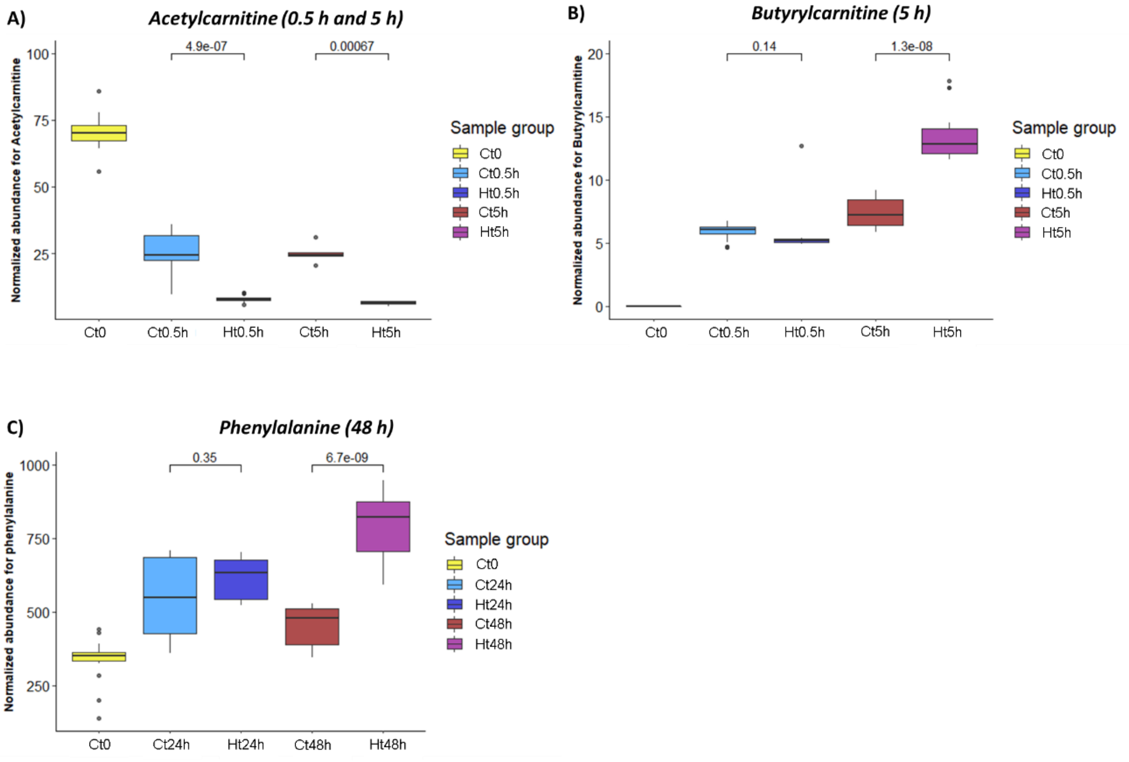
A)
Acetylcarnitine (0.5 h and 5 h)
Normalized abundance for Acetylcarnitine
100
75
50
25
4.9e-07
0.00067
Ct0
Ct0.5h
Ht0.5h
Ct5h
Ht5h
Sample group
Ct0
Ct0.5h
Ht0.5h
Ct5h
Ht5h
B)
Butyrylcarnitine (5 h)
Normalized abundance for Butyrylcarnitine
20
15
10
5
0
0.14
1.3e-08
Ct0
Ct0.5h
Ht0.5h
Ct5h
Ht5h
Sample group
Ct0
Ct0.5h
Ht0.5h
Ct5h
Ht5h
C)
Phenylalanine (48 h)
Normalized abundance for phenylalanine
1000
750
500
250
0.35
6.7e-09
Ct0
Ct24h
Ht24h
Ct48h
Ht48h
Sample group
Ct0
Ct24h
Ht24h
Ct48h
Ht48h

Figure S7

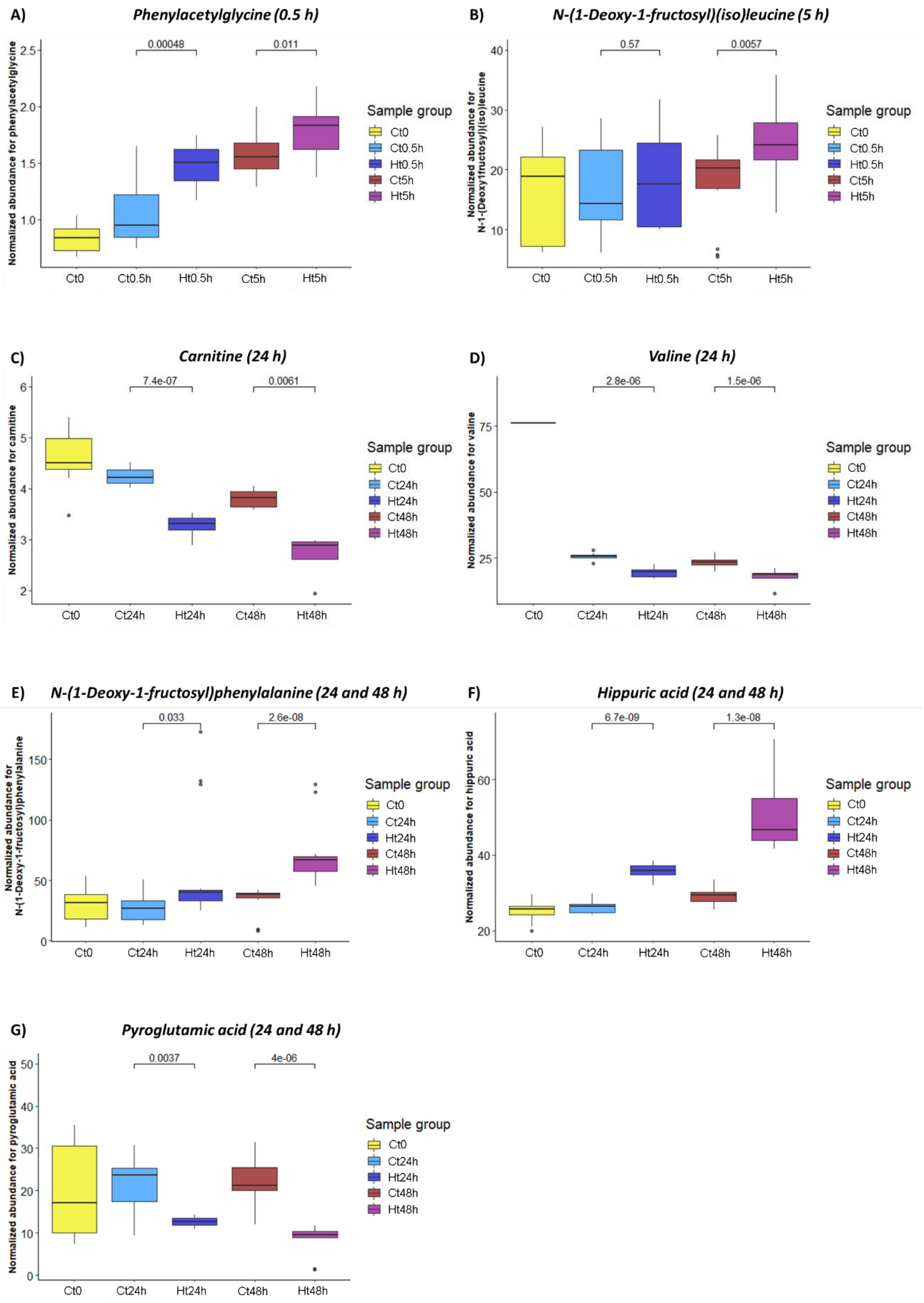
A)
Phenylacetylglycine (0.5 h)
Normalized abundance for phenylacetylglycine
0.00048
0.011
Ct0
Ct0.5h
Ht0.5h
Ct5h
Ht5h
Sample group
B)
N-(1-Deoxy-1-fructosyl)(iso)leucine (5 h)
Normalized abundance for N-1-(Deoxy1fructosyl)(iso)leucine
0.57
0.0057
C)
Carnitine (24 h)
Normalized abundance for carnitine
7.4e-07
0.0061
Ct0
Ct24h
Ht24h
Ct48h
Ht48h
D)
Valine (24 h)
Normalized abundance for valine
2.8e-06
1.5e-06
E)
N-(1-Deoxy-1-fructosyl)phenylalanine (24 and 48 h)
Normalized abundance for N-(1-Deoxy-1-fructosyl)phenylalanine
0.033
2.6e-08
F)
Hippuric acid (24 and 48 h)
Normalized abundance for hippuric acid
6.7e-09
1.3e-08
G)
Pyroglutamic acid (24 and 48 h)
Normalized abundance for pyroglutamic acid
0.0037
4e-06

Table S1. Unknown molecular features (identification level of 4 according to [19]). in the intracellular and extracellular fluid analysis at 0.5 and 5 h.

#	RT (min)	Monoisotopic mass (Da)	Main fragments (MS/MS)	VIP (Trend*)	
				**Ht0.5h *vs* Ct0.5h**	**Ht5h *vs* Ct5h**
**Intracellular fluid**					
**Significant metabolites at 0.5 h**					
1	2.9	113.0842	55.0537, 69.0685	1.30 (↑)	0.04 (↑)
2	1.1	161.0520	-	1.17 (↓)	0.94 (↓)
3	10.6	366.1278	160.0421, 217.0634	1.03 (↑)	0.13
**Significant metabolites at 5 h**					
4	0.7	103.1011	-	0.19 (↓)	1.09 (↓)
5	14.0	273.2696	-	0.73	1.59 (↓)
6	14.2	317.2957	88.0756, 57.0696, 70.0643	0.21	1.37 (↓)
7	16.2	301.3001	256.2625, 88.0756, 219.1733	0.05	1.24 (↓)
**Significant metabolites at 0.5 h and 5 h**					
8	1.7	219.1123	90.0547. 72.0439. 98.0230	1.21 (↓)	1.59 (↓)
9	7.6	760.1953	298.0556. 473.1246	2.51 (↑)	1.56 (↑)
10	8.0	308.1218	174.0576. 128.0519	1.05 (↑)	1.77 (↑)
11	8.1	352.1120	174.0578. 160.0421. 128.0525	1.83 (↑)	1.63 (↑)
12	9.4	455.1216	-	1.44 (↓)	1.35 (↓)
13	17.5	303.2956	91.0544. 58.0658	2.52 (↓)	2.19 (↓)
14	18.3	329.3319	87.0748. 70.0647. 106.0854	1.08 (↓)	1.63 (↓)
15	19.5	331.3270	91.0546. 58.0660	2.44 (↓)	2.33 (↓)
16	20.6	325.3745	186.2201	2.57 (↓)	2.32 (↓)
**Extracellular fluid**					
**Significant metabolites at 0.5 h**					
17	1.0	562.2164	-	1.46 (↑)	0.70 (↑)
18	7.1	415.0885	176.07139, 241.0301	1.80 (↑)	0.98 (↑)
19	7.1	240.0257	-	1.74 (↑)	0.93 (↑)
20	15.3	386.1751	105.0696, 121.0646	1.45 (↑)	0.43
**Significant metabolites at 5 h**					
21	0.9	85.0896	56.0478, 57.0555	0.26	1.01 (↓)
22	1.2	248.1280	29.1219, 174.1030, 189.1112	0.33	1.08 (↓)
23	1.3	349.1103	143.0296, 230.0785, 201.0580	0.35	1.30 (↑)
24	1.4	138.1147	83.0592	0.44	1.98 (↓)
25	6.3	480.2100	84.0805, 147.1162, 130.0862	0.00	2.52 (↑)
26	6.3	187.1216	56.0544, 83.0849, 97.1010	0.09	1.25 (↓)

Table S1 (continued)

#	RT (min)	Monoisotopic mass (Da)	Main fragments (MS/MS)	VIP (Trend*)	
				Ht0.5h *vs* Ct0.5h	Ht5h *vs* Ct5h
27	7.1	256.0974	186.1047	0.55 (↓)	1.27 (↓)
28	7.5	159.0359	87.0257, 70.0641, 114.0353	0.00	1.31 (↑)
29	7.7	606.1010	-	0.00	1.45 (↑)
30	8.2	453.1583	160.0413, 277.1199	0.00	1.2 (↑)
31	8.9	453.1067	91.0548, 156.0852	0.00	1.5 (↑)
32	9.2	354.0587	261.0211, 273.0901	0.91 (↑)	1.02 (↑)
33	11.0	159.0358	114.0371, 87.0257	0.73	2.61 (↑)
34	11.0	1161.1360	-	0.84 (↑)	1.18 (↑)
35	14.3	289.2633	242.2479, 88.0750, 56.0493	0.29 (↑)	1.02 (↑)
36	14.6	449.3731	344.3134	0.43 (↑)	1.15 (↑)
**Significant metabolites at 0.5 and 5 h**					
37	1.0	296.0949	122.0707	2.29 (↓)	1.82 (↓)
38	1.1	159.0689	130.0648,143.0700, 115.0526	1.13 (↑)	2.30 (↑)
39	1.3	264.0709	120.0783, 175.0158	1.12 (↑)	2.15 (↑)
40	2.1	301.1897	-	1.58 (↑)	1.56 (↑)
41	2.9	113.0844	55.0538, 69.0697, 79.0539	1.99 (↑)	1.24 (↑)
42	4.9	179.0956	91.0527, 62.0591	1.14 (↑)	2.09 (↑)
43	5.7	239.1171	194.1175, 126.0534	1.79 (↑)	1.45 (↑)
44	5.9	554.2221	247.1024	1.80 (↑)	1.78 (↑)
45	6.5	508.2161	201.0982	2.67 (↑)	2.63 (↑)
46	6.6	480.2109	322.1757,147.1114, 84.0803	2.72 (↑)	2.77 (↑)
47	6.6	321.1699	84.0786, 130.0855, 147.1179	2.65 (↑)	2.69 (↑)
48	6.6	159.0358	114.0372, 87.0273	2.62 (↑)	2.62 (↑)
49	6.6	489.1730	156.0766, 331.1418	2.53 (↑)	2.18 (↑)
50	7.1	574.1303	416.09380, 241.0301	1.88 (↑)	1.07 (↑)
51	7.1	159.0358	114.0349, 87.0267	1.85 (↑)	1.03 (↑)
52	7.3	480.2097	84.0807, 130.0852	3.13 (↑)	2.83 (↑)
53	7.5	574.1280	241.0289,160.0402	2.83 (↑)	1.45 (↑)
54	7.5	480.1702	147.0756	2.38 (↑)	2.53 (↑)
55	7.9	439.1425	160.0412, 114.0364	2.55 (↑)	1.65 (↑)
56	8.1	409.1321	160.0418, 114.0371	2.46 (↑)	2.68 (↑)
57	8.3	242.0818	198.0626, 172.0862	1.15 (↑)	1.12 (↑)
58	18	356.2719	-	1.31 (↑)	1.22 (↑)
59	20.3	357.3628	88.0738, 54.0757, 71.0780	1.46 (↑)	1.24 (↑)

****↑:** The metabolite (on average) is more abundant in hypoxia condition; ↓: The metabolite (on average) is less abundant in hypoxia condition.

Table S2. Unknown molecular features (identification level of 4 according to [19]) in the intracellular and extracellular fluid analysis at 24 and 48 h.

#	RT (min)	Monoisotopic mass (Da)	Main fragments (MS/MS)	VIP (Trend)	
				Ht24h *vs* Ct24h	Ht48h *vs* Ct48h
**Intracellular fluid**					
**Significant metabolites at 24 h**					
1	8.1	217.2042	70.0646, 88.0754, 57.0699	1.17 (↑)	0.99 (↑)
**Significant metabolites at 48 h**					
2	0.7	161.1040	90.0545	0.25 (↑)	1.01 (↑)
3	0.9	131.0941	86.0963, 69.0698	0.32 (↑)	1.02 (↑)
4	8.1	352.1092	174.0578, 160.0424, 128.0524	0.56 (↓)	1.55 (↑)
5	11.3	245.2356	88.0757, 57.0701, 70.0654	0.90 (↑)	1.00 (↑)
**Significant metabolites at 24 and 48 h**					
6	8.6	261.2304	80.0757, 70.0656, 57.0701	1.11 (↑)	1.06 (↑)
7	13.8	273.2668	88.07533, 57.0700, 70.0650, 106.0858	1.30 (↑)	1.10 (↑)
8	14.0	317.2929	256.2630, 88.0756	1.21 (↑)	1.04 (↑)
9	16.0	301.2984	-	1.22 (↑)	1.16 (↑)
10	17.2	303.2930	91.0540, 58.0653, 212.2367	3.11 (↑)	1.90 (↑)
11	18.0	329.3297	88.0755, 57.0702	1.34 (↑)	1.36 (↑)
12	19.2	331.3244	91.0538, 58.0653, 240.2672	2.25 (↑)	1.93 (↑)
**Extracellular fluid**					
**Significant metabolites at 24 h**					
1	0.8	132.0223	87.0989, 70.0702	1.04 (↑)	0.73 (↑)
2	1.2	281.1115	150.0731	1.74 (↑)	0.00
3	1.6	446.2217	-	1.12 (↑)	0.41 (↑)
4	1.7	443.1282	-	1.02 (↑)	0.00
5	3.7	191.0577	-	1.12 (↑)	0.78 (↑)
6	4.2	245.1617	85.0279	1.07 (↑)	0.62 (↓)
7	6.1	246.1686	85.0289	1.05 (↑)	0.49 (↑)
8	6.2	466.1885	308.1602, 70.0645	2.35 (↑)	0.34 (↑)
9	6.2	307.1536	-	1.95 (↑)	0.75 (↑)
10	6.4	230.1733	-	1.17 (↑)	0.50 (↑)
11	6.4	321.1683	84.0811, 130.0891, 141.1048	1.49 (↑)	0.89 (↑)
12	6.4	480.2040	322.1754, 84.0808, 147.1121	1.57 (↑)	0.97 (↑)
13	6.8	485.1283	454.1079	3.28 (↓)	0.00 ()

Table S2. (continued)

#	RT (min)	Monoisotopic mass (Da)	Main fragments (MS/MS)	VIP (Trend)	
				Ht24h *vs* Ct24h	Ht48h *vs* Ct48h
14	7.3	452.3350	86.0966, 309.8652	1.18 (↑)	0.82 (↑)
15	7.8	439.1406	160.0429	2.54 (↑)	0.20
16	8.1	646.2235	315.0941	2.48 (↓)	0.00
17	8.3	1471.5847	-	1.07 (↑)	0.20
**Intracellular fluid**					
18	10.4	159.0349	114.0370, 87.0271	2.11 (↑)	0.84 (↑)
19	16.0	257.2717	62.0621, 174.9822	1.21 (↑)	0.33 (↑)
20	16.1	473.2521	60.0554	1.01 (↑)	0.66 (↑)
21	16.2	317.2923	270.2830, 88.0750	1.11 (↑)	0.60 (↑)
22	17.2	303.2928	91.0557, 212.2369	2.35 (↑)	0.00
23	18.0	329.3294	88.0757, 57.0700	1.37 (↑)	0.18 (↑)
24	18.2	345.3236	298.3080, 74.0568, 56.0478	1.17 (↑)	0.46 (↑)
25	18.2	528.5210	256.2639	1.57 (↑)	0.12 (↑)
26	18.7	399.3712	338.3439, 57.0681, 102.0951	1.58 (↑)	0.38 (↑)
27	19.0	399.3698	338.3389, 88.0738, 100.9248	1.32 (↑)	0.98 (↑)
28	19.9	556.5537	256.2631, 284.2921	1.20 (↑)	0.47 (↓)
29	26.0	330.2740	57.0705, 71.0840, 95.0817	1.47 (↑)	0.04 (↑)
30	26.7	530.2727	119.0852	1.08 (↑)	0.07
31	31.0	679.3579	-	1.71 (↑)	0.42 (↑)
**Significant metabolites at 48 h**					
32	0.5	262.1387	86.0956, 132.0998	0.73 (↑)	1.05 (↑)
33	0.5	595.2805	-	0.47	1.11 (↑)
34	0.8	244.1412	-	0.41	1.26 (↑)
35	0.9	175.0615	70.0627, 60.0529	0.67 (↑)	1.21 (↑)
36	0.9	153.0756	-	0.82 (↑)	1.33 (↑)
37	1.3	583.1634	210.0453	0.61	1.47 (↑)
38	1.3	593.1157	210.0518	0.05 (↓)	1.16 (↑)
39	1.3	330.1541	120.0802, 166.0910	0.84 (↑)	1.06 (↑)
40	1.3	367.1228	203.0509, 188.0679	0.28	1.44 (↓)
41	1.4	250.0642	137.0442	0.62 (↑)	1.08 (↑)
42	1.6	219.1096	-	0.94 (↑)	1.22 (↑)
43	1.7	246.1204	86.0947	0.87	1.42 (↑)
44	2.2	408.1789	188.0698	0.62	1.23 (↑)
45	2.2	242.0453	-	0.47	1.41 (↑)
46	2.2	226.0714	-	0.62	1.31 (↑)

Table S2. (continued)

#	RT (min)	Monoisotopic mass (Da)	Main fragments (MS/MS)	VIP (Trend)	
				**Ht24h *vs* Ct24h**	**Ht48h *vs* Ct48h**
47	2.3	287.1155	-	0.85 (↑)	1.33 (↑)
48	3.5	246.1372	146.0573, 188.0718	0.82	1.05 (↑)
49	3.5	257.0724	164.0642, 126.0526	0.38	1.30 (↑)
50	3.6	877.3352	107.04627, 182.0818, 165.0543	0.02	1.51 (↑)
51	3.7	329.1584	106.04820, 197.1235, 86.0935	0.24 (↑)	1.27 (↑)
52	4.3	135.0680	91.0538	0.60	1.27 (↑)
53	4.4	265.1310	85.0273	0.54 (↑)	1.55 (↑)
54	4.6	412.1338	136.0605, 281.0978, 162.0403	0.91 (↑)	1.33 (↑)
55	4.7	266.0904	120.0442	0.09	1.12 (↑)
56	5.5	239.1158	194.1179, 126.0536	0.32	1.14 (↑)
57	6.1	187.1204	83.0849, 97.1003, 55.0541	0.70	1.13 (↑)
58	6.1	334.0982	289.10003, 128.0521, 159.0895, 91.0534	0.87	3.13 (↑)
59	7.0	159.0352	114.0370, 87.0268	0.89	1.85 (↑)
60	7.0	578.1194	243.0216, 418.0825	0.00	1.76 (↑)
61	7.1	480.2039	84.0804, 147.1116, 130.0856	0.87	1.86 (↑)
62	7.4	159.0351	114.0385, 87.0245	0.00	2.44 (↑)
63	7.6	606.0944	448.0646, 273.0032	0.00	1.37 (↑)
64	7.7	330.1010	253.1240	0.46	1.18 (↑)
65	7.8	346.0747	-	0.28	1.33 (↑)
66	7.8	638.2195	331.1081	0.01	1.74 (↑)
67	7.8	616.2380	309.1263	0.28	1.56 (↑)
68	8.2	334.0983	217.0644, 202.0300	0.53	1.38 (↑)
69	8.2	393.1468	-	0.58	1.95 (↑)
70	8.8	453.1027	91.0534, 139.0849, 176.0679	0.82	1.72 (↑)
71	9.8	231.2189	70.0633, 106.0843, 85.0276	0.70	1.07 (↑)
72	11.3	245.2353	88.0751, 70.0650, 57.0701	0.86 (↑)	1.26 (↑)
73	11.3	201.2088	57.0681	0.90	1.44 (↑)
74	11.4	465.1926	160.0411, 132.1014, 86.0950	0.68	2.00 (↑)
75	11.8	333.2875	272.2540, 228.2313, 70.0644	0.29	1.03 (↑)
76	12.1	499.1768	166.0836, 160.0415	0.84	1.08 (↑)
77	12.6	259.2507	57.0677, 70.0651, 95.0761	0.99	1.66 (↑)
78	13.1	465.3079	-	0.73	1.14 (↑)
79	14.3	449.3712	344.3135	0.90 (↑)	1.25 (↑)
80	14.5	493.3977	388.3389, 344.3175	0.66 (↑)	1.14 (↑)
81	27.9	539.5277	256.2628, 311.2939, 284.2938	0.64	1.10 (↑)

Table S2. (continued)

#	RT (min)	Monoisotopic mass (Da)	Main fragments (MS/MS)	VIP (Trend)	
				**Ht24h *vs* Ct24h**	**Ht48h *vs* Ct48h**
82	30.1	595.5914	313.3260	0.45 (↓)	1.87 (↓)
**Significant metabolites at 24 and 48 h**					
83	0.7	166.0814	81.0450, 122.0709, 139.0994	1.55 (↓)	1.00 (↓)
84	0.7	382.1053	-	1.53 (↓)	1.65 (↓)
85	0.7	204.0556	-	1.13 (↑)	1.09 (↑)
86	0.8	282.9963	226.0389	1.37 (↑)	1.36 (↑)
87	0.9	386.0913	176.0641	1.18 (↑)	1.21 (↑)
88	0.9	284.1697	154.0840	1.04 (↑)	1.21 (↑)
89	1.0	248.1269	219.1219, 174.1019, 186.0999, 137.0764	2.09 (↓)	1.71 (↓)
90	1.6	482.1827	264.0794	1.17 (↑)	1.31 (↑)
91	1.7	231.1459	85.0273	2.04 (↑)	1.66 (↑)
92	1.9	301.1877	102.0903	1.53 (↑)	1.53 (↑)
93	2.2	201.0785	184.0762, 156.0800	1.06 (↑)	1.29 (↑)
94	2.2	187.0629	118.0642, 146.0591	2.07 (↑)	1.61 (↑)
95	2.3	177.1257	91.05667	1.30 (↑)	1.43 (↑)
96	2.4	260.1364	-	1.65 (↑)	1.51 (↑)
97	2.5	297.0890	136.0622	1.93 (↑)	1.01 (↑)
98	2.7	289.0945	244.1010, 126.0534, 110.0374	1.38 (↑)	1.02 (↑)
99	2.8	260.1353	86.0964, 132.1010	2.20 (↑)	1.24 (↑)
100	2.9	243.1464	-	1.21 (↑)	1.49 (↑)
101	3.7	245.1619	85.0280	1.46 (↑)	1.65 (↑)
102	3.8	294.1210	120.0805, 166.0870	2.35 (↑)	1.61 (↑)
103	4.0	104.0264	77.0378, 51.0223	1.14 (↑)	1.11 (↑)
104	4.7	441.1390	295.0925	1.05 (↑)	1.21 (↑)
105	5.1	75.0323	-	1.05 (↑)	1.69 (↑)
106	5.1	193.0748	-	1.27 (↑)	2.62 (↑)
107	5.7	1354.5665	-	1.14 (↑)	1.29 (↑)
108	6.2	480.2036	322.1745, 147.1118, 84.0802	1.93 (↑)	1.41 (↑)
109	6.3	508.2099	201.0954, 175.1178	1.66 (↑)	1.33 (↑)
110	6.4	159.0353	114.0369	1.54 (↑)	1.13 (↑)
111	6.4	489.1673	156.0765, 110.0704	1.46 (↑)	1.69 (↑)
112	7.0	508.2101	201.0950, 175.1193	1.51 (↑)	2.11 (↑)
113	7.0	415.0869	241.0339, 151.9829, 122.0254	2.80 (↑)	1.80 (↑)
114	7.0	574.1220	416.0927, 241.0300	3.62 (↑)	2.34 (↑)
115	7.4	574.1221	416.0932, 241.0298	2.76 (↑)	2.98 (↑)

Table S2. (continued)

#	RT (min)	Monoisotopic mass (Da)	Main fragments (MS/MS)	VIP (Trend)	
				**Ht24h *vs* Ct24h**	**Ht48h *vs* Ct48h**
116	7.4	415.0871	241.0300, 176.0705, 122.0309, 161.9846	2.08 (↑)	2.39 (↑)
117	7.7	670.1566	289.0257, 363.0442, 192.0071	1.16 (↓)	1.61 (↑)
118	7.9	481.1533	160.0424, 148.0622	1.26 (↑)	1.10 (↑)
119	8.0	409.1304	160.0423, 176.0694	2.25 (↑)	1.06 (↑)
120	8.1	453.1563	160.0438, 277.1150, 129.1042, 95.2478	2.12 (↑)	1.53 (↑)
121	8.2	439.1410	160.0435	1.16 (↑)	1.67 (↑)
122	8.3	1605.6796	-	1.27 (↑)	1.09 (↑)
123	8.4	423.1459	160.0422, 176.0682	1.85 (↑)	1.36 (↑)
124	8.8	449.1615	116.0703, 160.0420	1.35 (↑)	2.00 (↑)
125	10.4	424.1300	160.0420, 217.0634	4.03 (↑)	3.23 (↑)
126	10.9	393.1462	60.0553	2.72 (↑)	2.83 (↑)
127	11.4	357.3597	-	1.35 (↑)	1.55 (↑)
128	11.8	319.2872	91.0536, 228.2333, 58.0651	2.98 (↑)	2.64 (↑)
129	11.9	319.2874	91.05307, 58.0649, 228.2335	3.03 (↑)	2.40 (↑)
130	13.8	273.2667	88.0753, 57.0698, 70.0653, 106.0862	1.19 (↑)	1.47 (↑)
131	13.8	229.2404	57.0700	1.06 (↑)	1.38 (↑)
132	14.0	317.2931	-	1.01 (↑)	1.59 (↑)
133	14.1	361.3192	300.2883, 70.0649	1.31 (↑)	2.06 (↑)
134	14.2	405.3450	344.3126, 300.2946	1.08 (↑)	1.41 (↑)

****↑:** The metabolite (on average) is more abundant in hypoxia condition; ↓: The metabolite (on average) is less abundant in hypoxia condition.

Table S3. Cell number and protein content of the sample groups at short times.

Sample group	Ct0	Ct0.5	Ct5h	Ht0.5	Ht5h
**Cell number**	180000	162000	161000	14900	153000
**Protein content (mg/500μL)**	0.286	0.291	0.248	0.228	0.242

Table S4. Cell number and protein content of the sample groups at long times.

Sample group	Ct0	Ct24h	Ct48h	Ht24h	Ht48h
**Cell number**	221000	220000	224000	217000	180000
**Protein content (mg/500μL)**	1.076	1.062	1.130	1.085	0.984
